# Insulin-Like Growth Factor-2 Is Induced Following 5-Aminolevulinic Acid-Mediated Photodynamic Therapy in SW620 Human Colon Cancer Cell Line

**DOI:** 10.3390/ijms161023615

**Published:** 2015-10-02

**Authors:** Marta Woźniak, Kamila Duś-Szachniewicz, Piotr Ziółkowski

**Affiliations:** Department of Pathology, Wroclaw Medical University, Wrocław 50-368, Poland; E-Mails: marta1wozniak@wp.pl (M.W.); kamila.dus@gmail.com (K.D.-S.)

**Keywords:** photodynamic therapy, insulin-like growth factor-2, insulin-like growth factor-2 receptor, insulin-like growth factor-2 binding protein-1

## Abstract

The IGF system is a family of polypeptide growth factors, which plays a significant role in the development and growth of many cells. Dysregulation of insulin-like growth factors and their pathway components has been connected with essential tumor properties, such as tumor cell proliferation, antiapoptotic properties, invasive behavior and chemotherapy resistance. However, the effects of photodynamic therapy (PDT), one of the cancer treatment methods for the regulation of the IGF signaling pathway, are still unclear. The aim of this study was to investigate the expression of IGF-2 after 5-aminolevulinic acid (5-ALA)-mediated-PDT in SW620 human colorectal cancer cells with evaluation of cell proliferation and apoptosis and to determine the effects of PDT on the IGF-2 receptor (IGF-2R), IGF-2 binding protein-1 (IGF-2BP-1) and the proapoptotic protein, BAX. Cells were treated with 5-aminolevulinic acid and its methyl ester. Changes of the expression and concentration of IGF-2 before and after treatment were assayed by immunocytochemistry, Western blot and ELISA. We found that IGF-2 was significantly overexpressed in the SW620 cell line, while its receptor and binding protein-1 were not significantly changed. Within this study, we would like to suggest that IGF-2 contributes to the effects of PDT and that its expression will influence post-PDT efficacy.

## 1. Introduction

Colorectal cancer is the third most frequently-diagnosed malignant tumor worldwide (1.23 million, 9.7% after lung and breast cancer), and its incidence has been increasing in recent years [1]. Obesity, smoking, physical inactivity, red and processed meat consumption and excessive alcohol consumption are clear risk factors that promote the development of the colon cancer [2].

What is important is that an expected decrease in the risk of colon cancer is associated with improved treatment, increased awareness and its early detection. Conventional chemotherapeutic agents, although often effective, are highly toxic because of their lack of selectivity for cancer cells. As a result, efforts have been focused on other methods based on the construction of tumor-selective treatment [3].

The photodynamic method known as photodynamic therapy (PDT) is widely used as a diagnostic method and mode of treatment without the above-mentioned drawback. It comprises three agents: a light-sensitive drug, visible light at the appropriate wavelength, reactive oxygen species or singlet oxygen. After irradiation, cells undergo destruction through programmed cell death [4]. In the present study, 5-aminolevulinic acid (5-ALA) and its methyl ester (met-ALA) were used as precursors of the light-sensitive naturally-occurring dye, protoporphyrin IX, in low doses. Following low-dose light irradiation (4.5 J/cm^2^, 50 mW/cm^2^), the expression of insulin-like growth factor-2 (IGF-2) system components was evaluated in the SW620 colon cancer cell line.

Insulin-like growth factor-1 and -2 (IGF-1, IGF-2), also called somatomedin C and A, respectively, are small ligands (~8 kDa) that promote cell growth and prevent cells from dying. They are structurally similar to insulin. In the human body, both IGF-1 and IGF-2 are produced in multiple tissues throughout the entire life [5]. IGF-1 is used across the lifespan of a human organism, but becomes most prominent during childhood and adolescence [6]. IGF-2 encourages cell proliferation, growth and metabolism in a variety of tissues. It also prepares the uterus for pregnancy and causes embryonic growth after conception [7].

IGF-2 bioavailability is regulated by membrane surface receptors. The IGF-1 receptor belongs to receptor tyrosine kinase (RTK) class II, and the IGF-2 receptor is the mannose-6-phosphate receptor (IGF-2/M6PR, IGF-2R). The IGF-1 receptor binds both IGF-1 and IGF-2, whereas the IGF-2/M6P receptor is primarily responsible for clearing away IGF-2 molecules, thereby reducing the levels of IGF-2 and acting as a signaling antagonist, preventing IGF-2 responses [8].

The IGF-2 signaling mechanisms through IGF-1R may promote cancer development by activation of the PI3-K/Akt and MAP kinase pathway. This process leads to a reduction of apoptosis and an increase of cell survival by inhibition of programmed cell death [9].

Chemical resemblance to insulin allows both IGFs to activate also the insulin receptor (IR). This receptor has two isoforms: IR-A and IR-B. The latest studies show that IR-A actually has even a higher affinity for IGF-2 than it does for insulin [9].

The effects of IGFs can be modulated by six proteins, called insulin-like growth factor binding proteins (IGF-BPs 1–6). They are circulating transport proteins for IGF-1 and IGF-2; IGF-BPs plays various roles in the circulation, the extracellular environment and inside the cell [10].

Abnormal levels of IGF-2 are thought to be associated with a rare condition causing excessive growth in the fetus. Moreover, dysregulation of the IGF signaling system partly triggers the underlying mechanism of uncontrolled increase in cellular proliferation, and it is involved in the development of certain cancers [11]. Many studies are focused on the specific mechanisms involving IGF system members.

The effects of photodynamic therapy on the regulation of the IGF signaling pathway are still unclear. The aim of this study was to determine the effects of oxidative stress caused by low dose 5-ALA and its methyl ester mediated PDT to IGF-2, the IGF-2 receptor (IGF-2R) and IGF2-BP-1 expression in the SW620 colon cancer cell line, additionally combined with the evaluation of apoptosis after PDT, including immunohistochemical studies on BAX protein. We aimed at checking whether a low dose PDT may induce the expression of these proteins, which in the light of the above-mentioned IGF functions could be an undesirable effect.

## 2. Results and Discussion

### 2.1. Cell Proliferation MTT Assay

**Figure 1:**
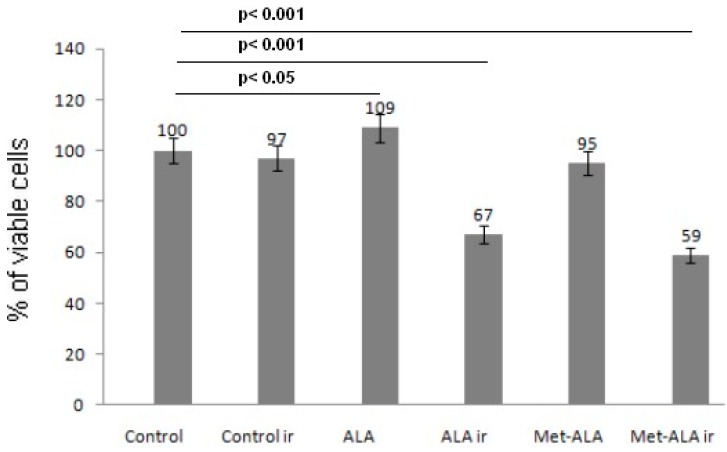
Percent of viable cells determined by the MTT assay. Photodynamic therapy by means of 5-aminolevulinic acid (5-ALA) or its methyl ester (met-ALA) decreased the viability of SW620 cells. Cells were treated with equal concentration of the above precursors for 4 h (5-ALA, Met-ALA) and then irradiated with 4.5 J/cm^2^ at 630 ± 20 nm (ir, irradiation). Cell viability was measured after 24 h following irradiation. All data are the means in percent with 5% statistical error from three experiments.

To identify the best concentration of photosensitizer precursor for low dose photodynamic therapy (cytotoxicity over 40% after irradiation), various levels of 5-ALA and met-ALA were cultured within cancer cells in the preliminary study (initial density 4 × 10^4^ cells/well, 48-well plate). Viability was confirmed using the MTT assay. The SW620 cell line treated with 3 mM of either 5-ALA or met-ALA did not cause the loss of cell proliferation. Cells incubated with 5-ALA only showed a slight significant increase of cell proliferation (*p* = 0.043), while cells treated with met-ALA insignificantly reduced their viability (up to 95%); whereas the cell survival rate after irradiation with 4.5 J/cm^2^ decreased significantly (*p* < 0.05) up to 67% for 5-ALA and 59% for met-ALA (Figure 1). For the next experiments, 3 mM of photosensitizer precursors were used.

### 2.2. Immunocytochemistry

**Figure 2:**
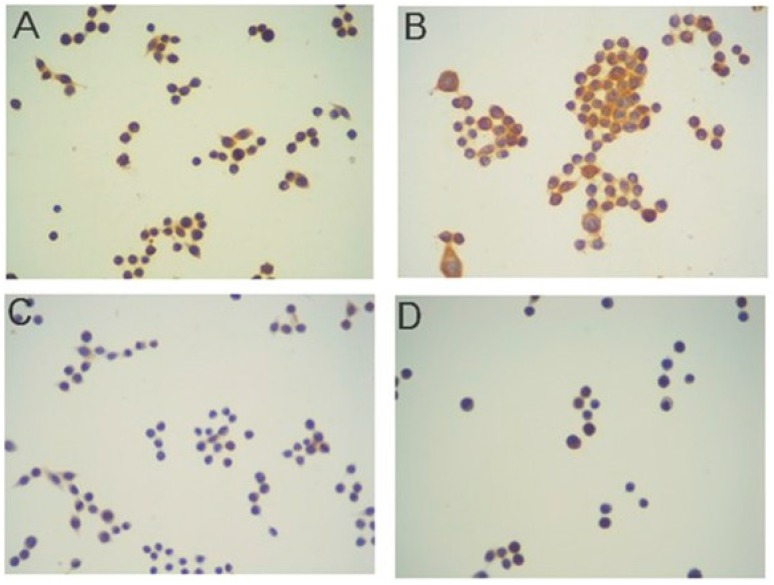

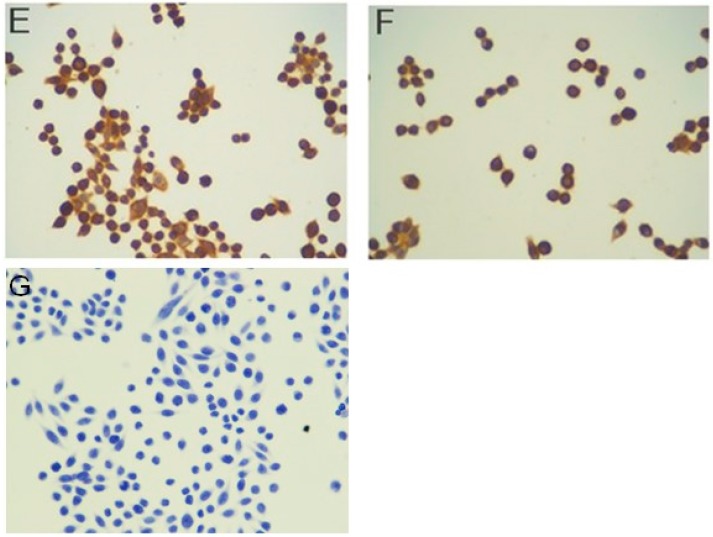
Immunostaining results in the SW620 human colon adenocarcinoma cell line. DAB method, hematoxylin-counterstained; magnification: 100×. IGF-2 staining. Cells were exposed to 3 mM 5-ALA for 4 h without light (**A**) and then treated with light (**B**); IGF-2 receptor: cells were exposed to 3 mM 5-ALA for 4 h without a light (**C**) and with light (**D**); IGF-2BP-1 staining: cells were exposed to 3 mM 5-ALA for 4 h without light (**E**) and with light (**F**); negative control (**G**).

The major finding in our study was that following photodynamic therapy, IGF-2 was expressed in most of the SW620 human colon cancer cells. Figure 2A shows the effects of immunostaining in control cells, which were exposed to 3 mM 5-ALA for 4 h without light and stained with antibody to IGF-2. It is clearly visible that some cells present a positive staining (immunoreactivity score, IRS = 4). However, after the PDT, the number and intensity of stained cells significantly increased to nine (IRS = 9), (Figure 2B). Figure 2C,D shows the effects of immunostaining in SW620 cancer cells before and after the PDT with regard to the IGF-2 receptor, respectively. The number of stained cells was very low, and the intensity of staining was very weak in both conditions, *i.e.*, before PDT and after, the immunoreactivity score amounted to only two. This low level of protein expression was observed throughout the entire study; the IGF-2R remained low also after PDT (IRS = 2). In contrast, the immunostaining of IGF-2-BP-1 showed a very high rate (IRS = 9) of immunoexpression in both pre- and post PDT cases. Figure 2E,F shows the expression of that binding protein in SW620 colon cancer cells treated with 3 mM 5-ALA only and PDT, respectively. This expression was very high and noticeable in cell cytoplasm, similarly to the pattern of staining with regard to IGF-2 and IGF-2R, where it was also cytoplasmic.

**Figure 3:**
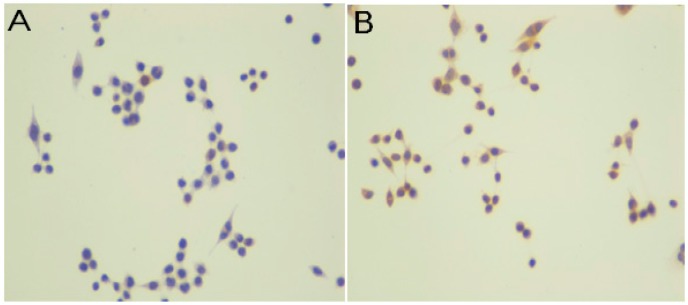
Immunostaining results in the SW620 human colon adenocarcinoma cell line for BAX protein. DAB method, hematoxylin-counterstained; magnification 100×. (**A**) Cells were exposed to 3 mM 5-ALA for 4 h without light and stained with antibody to BAX. No staining was observed in this case (immunoreactivity score (IRS) = 0); (**B**) following the PDT (3 mM 5-ALA and 4.5 J/cm^2^ light), strong expression of BAX was seen in cell cytoplasm (IRS = 6).

Figure 3 shows the effect of 5-ALA-PDT on SW620 cancer cells in relation to BAX protein. Figure 3A, before the PDT, shows a very weak expression of BAX in less than 5% of cells (IRS = 0), whereas after the PDT, the level of protein expression largely increased to six in the immunostaining reactivity score (IRS = 6) (Figure 3B).

All studies performed by means of 5-ALA and its methyl ester (met-ALA) showed a very similar pattern of staining with regard to both the number of stained cells and the intensity of staining (data for met-ALA are therefore not shown).

### 2.3. Western Blot Analysis

**Figure 4:**
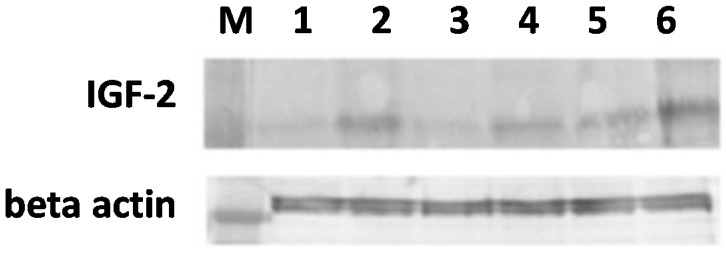
Western blot analysis of IGF-2 protein in the SW620 human colon adenocarcinoma cell line. Cells were exposed to 3 mM 5-ALA or met-ALA and additionally treated with light (4.5 J/cm^2^) or not irradiated. Differences between samples were found: Lane 1 shows the band from control cells without treatment; Lane 2 from cells treated with light only; Lanes 3 and 5 from cells incubated with 5-ALA or met-ALA, respectively; Lanes 4 and 6 from cells treated with PDT at the above doses. An increase in IGF-2 was also observed in cells treated with light only (Lane 2) and photosensitizer precursors without light irradiation (Lanes 3 and 5). M, Marker mass.

Western blot analysis of cell lysates of SW620 cells at relevant conditions (control cells pre-PDT *vs.* cells post-PDT) confirmed the changes of IGF-2 expression observed in immunocytochemical studies (Figure 4). We observed a strong difference between samples incubated with 5-ALA or met-ALA and irradiated (Lanes 4 and 6, respectively) and control cells without a treatment (Lane 1). An increase in IGF-2 was also observed in cells treated with light only (Lane 2) and photosensitizer precursors without light irradiation (Lanes 3 and 5).

### 2.4. Apoptosis Detection

**Figure 5:**
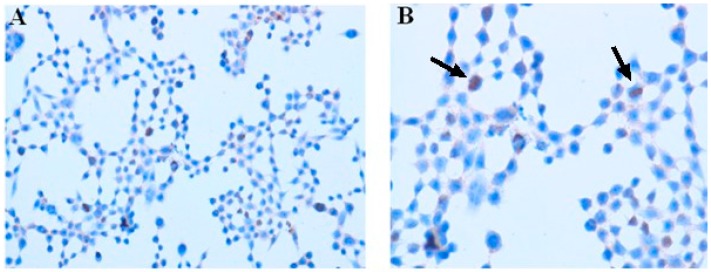
Apoptosis detection assay in the SW620 human colon adenocarcinoma cell line by means of immunocytochemistry. (**A**) Cells were exposed to 3 mM 5-ALA and additionally treated with light (4.5 J/cm^2^); (**B**) peroxidase *in situ* TUNEL method, hematoxylin-counterstained. Apoptosis was recognized due to the increased number of apoptotic bodies, shown by the arrows. Magnification: (**A**) 100× and (**B**) 200×.

5-ALA-PDT (3 mM 5-ALA, 4.5 J/cm^2^) resulted in an increase of apoptosis. The apoptotic bodies were detected by the TUNEL method (arrows in Figure 5). The number of TUNEL-positive cells was counted and presented as a percentage of apoptotic cells in relation to cells in the slide. We observed apoptosis induction following the PDT (>15%) in comparison to control cells (no precursor, no light: <5%). A similar pattern of staining was observed with regard to cells treated with met-ALA-PDT (data not shown).

### 2.5. ELISA Test

**Figure 6:**
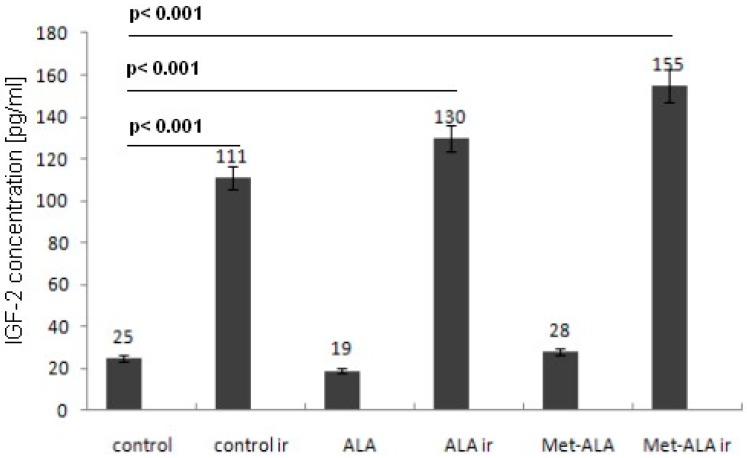
Results from the ELISA test performed on SW620 human colon cancer cells exposed to 3 mM 5-ALA or met-ALA and 4.5 J/cm^2^ light or left without light treatment and in control cells (no precursor, no light) or light only. A significant increase in IGF-2 concentration was found after photodynamic therapy (PDT) and light only (*p* < 0.05). Data are presented as the means with 5% statistical error from two experiments; ir, irradiation.

This study showed strong differences in the concentration of IGF-2 in pg/mL between control samples (including cells treated with 5-ALA or met-ALA only) and cells treated with the PDT. A significant increase in IGF-2 concentration was also found in cells exposed to light treatment only (Figure 6).

### 2.6. Discussion

Our previous studies showed that PDT results in the induction of several proteins, such as nuclear ubiquitous casein and cyclin-dependent kinases substrate (NUCKS), a protein that recently gained much attention as playing a role in tumor growth and showing some features of a tumor biomarker [12,13,14]. Next, we found the expression of two other proteins, c-Src kinase and c9orf10 protein, also called Ossa (C9orf10, Homo sapiens chromosome 9 open reading frame 10), following exposure to PDT [15]. The aim of the present study was to evaluate IGF-2, IGF-2R, IGF-2BP-1 and BAX expression following PDT *in vitro*. We investigated the effect of photodynamic therapy in the regulation of the IGF-2 system levels in comparison to the endogenous level in colon adenocarcinoma cell line SW620. We used low dose PDT to prevent cancer cells from 100% death.

The IGF signaling pathway is of central relevance in embryogenesis, as well as lifespan regulation and exhibits potent proliferative and antiapoptotic properties. Key regulatory molecules of this axis are small ligands (IGF-1 and IGF-2, 8 kDa), IGF binding proteins (IGF-BP1–6) and membrane-bound receptors (IGF-1 receptor (IGF-1R)), as well as the mannose-6-phosphate receptor (M6P/IGF-2R). The M6P/IGF-2R functions as a growth suppressor gene in colorectal cancer cells and provides dynamic *in vitro* functional support for the hypothesis that it is a human growth suppressor gene [16]. However, the system is far more complex, and its dysregulation results in essential tumor properties, such as tumor cell proliferation, antiapoptosis neo-angiogenesis, invasive behavior and chemotherapy resistance [11]. An important finding is that IGF-2 was more frequently detected in primary tumors, e.g., in human breast cancer cell lines, than IGF-1 [17]. Therefore, we focused on IGF-2 and its receptor in the present study.

In our studies, dysregulation of *IGF-2*, its receptor and IGF-BPs through oxygen stress provides part of the underlying mechanism for the uncontrolled increase in its expression in cell and cellular supernatant, even after decreased proliferation, as is evident in cancer cells after PDT treatment.

In the present study, we observed a significant increase in the protein expression of insulin-like growth factor-2 (IGF-2), which was accompanied by an increase in apoptosis, as measured by elevated BAX expression following PDT *in vitro*. The increased expression of BAX following PDT was commonly reported in previous studies [18]. Western blot analysis revealed that the transfected cell line showed overexpression of both Bcl-2 and BAX and that PDT caused selective destruction of Bcl-2, leaving BAX unaffected. The greater apoptotic response by the transfected line was, therefore, attributed to the higher BAX:Bcl-2 ratio after photodamage [19].

Our study did not show any change in the expression of insulin-like growth factor-2 receptor (IGF-2R) and IGF-2-binding protein-1. Whereas the expression of IGF-2-BP1 was very high before and after the PDT, the expression of IGF-2R remained low in all examined time points, both before and after the treatment. High, and increasing, serum levels of IGF-2-BP-3 were previously connected to decreased prostatic cancer risk [20]. Presumably, IGF-2-BP-1 might play a similar role in decreasing the potential of malignant cells.

Frequent loss of heterozygosity at the *M6P/IGF-2R* locus has been reported for many human cancers. The known effects of IGF-2R comprise its documented role as a growth inhibitor, and loss of its function is associated with progression of tumorigenesis and, thus, of a cancer [21,22]. Past data implicated abnormal M6P/IGF-2R-mediated growth control in carcinogenesis also involving colorectum, stomach and endometrium, but not the pancreas [23]. The loss of M6P/IGF-2R in cancer cells may lead to several negative phenomena, like tissue invasion and metastasis, by degrading basement membrane and extracellular matrix components [24]. The low expression level of IGF-2R observed in our study could therefore be another undesirable effect of low dose PDT besides increased IGF-2 expression. Decreased expression of IGF-2R enhanced IGF-2-induced proliferation and reduced susceptibility to tumor necrosis factor-induced apoptosis [25]. In our study, we found high expression of BAX protein, both pre- and post-PDT, which could neutralize the effects of IGFs and IGF-Rs through the induction of apoptosis.

The findings provide evidence that PDT can lead to an increase in IGF-2 protein expression. It is a well-known fact that the presence of IGF-2BP-1 can inhibit IGF-2 activity by decreasing the levels of free IGF-2 available to activate the receptor. Thus, high pre- and post-PDT expression of IGF-2BP-1 can play an important role in reducing the known effects of IGF-2, which can protect, e.g., breast cancer cells from cell death routinely induced by radiation and chemotherapy [26].

Moreover, IGF-2 bioavailability may also in part be regulated through IGF-2R; downregulation or deletion of this receptor should theoretically lead to locally-increased concentrations of IGF-2 molecules. Our study showed a significant increase in IGF-2 expression with concomitant low expression of its receptor, and therefore, despite the observed high level of IGF-2BP-1, this effect could presumably limit the efficacy of PDT *in vitro*.

## 3. Experimental Section

### 3.1. Precursors of Photosensitizer

In the experiments, 5-aminolevulinic acid and its methyl ester (Sigma–Aldrich, Munich, Germany) were used in one concentration of 3 mM in all experiments.

### 3.2. Cell Line

Studies were performed on the SW620 human colon adenocarcinoma cell line purchased from the Institute of Immunology and Experimental Therapy, Wrocław, Poland.

### 3.3. Light Source

All illuminations were performed using a halogen lamp (Penta Lamps, Teclas, Switzerland) at an excitation wavelength of 630 ± 20 nm selected with a band pass filter. The total light dose was 4.5 J/cm^2^ and irradiance 50 mW/cm^2^ after 4 h incubation time with the precursor of the photosensitizer.

### 3.4. Cell Proliferation MTT Assay

Cell proliferation and concomitantly PDT cytotoxicity before Western blot and immune-cytochemistry analysis were assessed via the 3-(4,5-dimethylthiazol-2-yl)-2,5-diphenyltetrazolium bromide assay (MTT assay), which is based on the reduction of a yellow soluble tetrazole to an insoluble purple formazan in respiring cells. Cells were seeded at an initial density of 4 × 10^4^ cells/well in a 48-well plate and grown. Then, control cells and cells treated with the precursor of the photosensitizer and its methyl ester were irradiated with light. After 24 h from irradiation, the medium was removed, and the cells were washed with phosphate-buffered saline (PBS). One milliliter of MTT/phenol-red free DMEM/F12 (*v*/*v* = 1:9) was added to each well, and the cells were incubated for 4 h at 37 °C. The medium was removed carefully, and 100 μL of dimethyl sulfoxide (DMSO, Sigma–Aldrich) were added to each well to solubilize the result of MTT, for 5 min. The optical absorbance (*A*) was measured at 490 nm using a BioTek ELX800 multi-well reader (BioTek, Winooski, VT, USA). The absorbance in the control group was regarded as 100% cell viability. The percentage of viable cells (VC) was calculated according to: VC (%) = (*A* of experimental group/*A* of control group) × 100.

### 3.5. Cell Culture Conditions

The colon adenocarcinoma SW620 cell line was cultured in DMEM/F12 (Gibco, Life Technologies, Carlsbad, CA, USA) supplemented with 10% FBS and 1% glutamine (GlutaMax, Life Technologies, Carlsbad, CA, USA). Cells were incubated at 37 °C in a 5% CO_2_ and 95% humidified atmosphere and kept in the logarithmic growth phase. The cells were seeded in 48-well and 12-well culture plates (TPP, Trasadingen, Switzerland) at a density of 4 × 10^4^ and 12 × 10^5^ cells/well, respectively. Cells were counted in suspensions using a Countess Automated Cell Counter (Invitrogen, Darmstadt, Germany). Then, the cells were cultured for the next 48 h. The medium was replaced, and cells were incubated with DMEM/F12 with 5-ALA (3 mM) or met-ALA (3 mM) for 4 h. After incubation, the medium was replaced with FBS-free DMEM/F12, and the irradiation was carried out with red light. After exposure to light, the medium was changed again into normal DMEM/F12.

Each experiment was carried out in triplicate, and groups of cells comprised cells treated with PDT, treated with precursors only, treated with light only or left without a treatment.

### 3.6. Cells Culture and Photosensitization Conditions in Experiments for Immunocytochemical Studies

Cells were seeded on 3-well (each well with a diameter of 14 mm), epoxy-coated, diagnostics glass slides (Menzel-Glaser, Braunschweig, Germany), 4 × 10^4^ cells per well, placed into humidified Petri dishes, grown and treated in the same conditions as described in Section 3.8.

### 3.7. Immunocytochemistry

The experiment was stopped after 24 h following light exposure when cells for immunocytochemistry (ICC) were placed in 4% paraformaldehyde at 4 °C for 10 min and then washed with PBS (0.1 M phosphate buffer, pH 7.4, 0.15 M NaCl). Immunocytochemistry was performed as previously reported using the LSAB+ method (LSAB+ System HRP from DAKO, Glostrup, Denmark). Glass slides with cells were incubated with endogenous peroxidase blocking buffer and then were incubated with protein blocking buffer. Next, primary antibodies against examined proteins (anti-IGF-2, IGF-2 receptor, IGF-2BP-1 and BAX, Sigma–Aldrich, dilution 1:100) were used, and slides were stored overnight at 4 °C. The next day, the slides were incubated for 15 min with each of the following two reagents: biotinylated link antibody and streptavidin-HRP with rinsing twice with PBS between and after and stained with 3,3’-diaminobenzidine in chromogen solution. Finally, sections were counterstained with Mayer’s hematoxylin and then dehydrated in graded alcohols, cleared in xylene and mounted with xylene-based mounting medium. The negative control was obtained by omitting the first antibody. Photographs were taken by light microscope fitted with a digital camera (Nikon Eclipse 80i with camera DS-Fil-U2, Amsterdam, The Netherlands) at magnifications of 100× and 200×.

The protein expression of IGF-2-, IGF-2R- and IGF-2BP-1-positive cells in the glass slide was defined as the number of total counts of intensity and quantity in the immunoreactivity score (IRS), as shown in Table 1. The same method was applied to the evaluation of histological slides with staining against BAX protein.

**Table 1:** The immunoreactivity score (IRS) calculation method. The final score was calculated by multiplying the staining intensity value by the percentage of staining.

Intensity of Staining	Number of Stained Cells (Quantity)
0, negative staining	0, <5%
1, weak	1, 5% to 25%
2, moderate	2, 25% to 75%
3, strong	3, >75%

The final score of immunoreactivity ranged then from 0 to 9. The glass slides were evaluated by two independent pathologists in a blind manner using a light microscope (Nikon Eclipse 80i with camera DS-Fil-U2) at 100× magnification. Their validation was in agreement in the case of 85% of the slides; any disagreement was discussed, and then, slides were classified into the most adequate category of IRS.

### 3.8. Cell Culture and Photosensitization Conditions in Experiments for Western Blot Analysis

For experiments, the cells were seeded on plastic 12-well plates (12 × 10^5^ cells/well). The cells were incubated with the precursor of the photosensitizer (5-ALA or its methyl ester) for 4 h in the medium with fetal bovine serum reduced to 5% and without phenol red. Next, the medium was replaced by the medium without serum and phenol red, and then, it was exposed to the proper light. After irradiation, the medium was replaced again by full DMEM/F12. The experiment was stopped 24 h after irradiation by collecting the medium, double washing of the cells with PBS and lysing the cells.

### 3.9. Western Blotting

For the Western blot analysis, cells were washed twice with pre-cooled PBS and, subsequently, were treated with lysis buffer (4% SDS, 0.1 M DTT, in 0.1 M Tris–HCl buffer pH 7.6, 200 μL/well) containing protease and phosphatase inhibitors (1% cocktails, Sigma–Aldrich). The lysates were cleaned by centrifugation at 15,000× *g* for 30 min. The supernatant was collected, and the protein concentration was measured at 280 nm using a spectrophotometer, PicoDrop 2000 (ThermoFischer Scientific, Inc., Waltham, MA, USA). Total protein extracts were separated on 4% to 12% SDS-PAGE (sodium dodecyl sulfate polyacrylamide gel electrophoresis, equipment from Invitrogen, Carlsbad, CA, USA) and transferred to the nitrocellulose (AmershamHybond, Healthcare Bio-sciences AB, Uppsala, Sweden). The membrane was blocked with phosphate-buffered saline containing 0.1% Tween 20 (pH 7.6 with 10% goat serum (Sigma–Aldrich) for 1 h at room temperature. Subsequently, the membrane was incubated overnight at 4 °C with the first antibodies’ solution (diluted 1:100). The primary antibodies used in this study included anti-IGF-2, IGF-2 receptor and IGF-2BP-1, and they were all purchased from Sigma–Aldrich. After washing twice with PBS, the membrane was incubated with horseradish peroxidase-labeled secondary goat anti-rabbit antibody (Santa Cruz Biotechnology, Inc., Santa Cruz, CA, USA) for 1 h at room temperature and thereafter washed three times with PBS. The final detection was performed with enhanced colorimetric Western blotting visualization reagents using the DAB Enhanced Liquid Substrate System for Immunochemistry (Sigma–Aldrich). The results were documented using appropriate Bio-Rad equipment (Molecular Imager Gel Doc TMXR+, BioRad, Hercules, CA, USA). Loading differences were normalized by using a monoclonal β-actin antibody (Santa Cruz Biotechnology, Inc., Santa Cruz, CA, USA) against the housekeeping control β-actin.

### 3.10. IGF-2 ELISA

For detection of IGF-2, the cell culture supernatants were collected from cells prepared in the same way as described in Section 3.8. The concentrations of IGF-2 in the supernatants were detected by the Human IGF-2 ELISA kit (Insight Genomics, Booster Immunoleader, Sterling, VA, USA). This kit is based on the standard sandwich enzyme-linked immune-sorbent assay technology. The purified anti-IGF-2 antibody was pre-coated onto 96-well plates, and the biotin conjugated anti-IGF-2 antibody was used for the detection of antibodies. Supernatants were centrifuged to remove particles and stored in −20 °C. One hundred microliters of each sample were used for the assay to quantify IGF-2, as specified by the manufacturer’s instructions. The standards, test samples and biotinylated antibody were added to the wells subsequently, mixed and incubated; then, unbound antibody was washed away with wash buffer. TMB substrates were used to visualize the enzymatic reaction with avidin–biotin–peroxidase complex (ABC) solution. This blue-colored product changed into yellow after adding TMB stop solution. The density of yellow is proportional to the IGF-2 amount of sample captured in the plate. The OD absorbance at 450 nm in a microplate reader (BioTek, Winooski, VT, USA) was read. The concentration of IGF-2 in samples was calculated from a standard curve generated each time the assay was performed. The experiment was repeated twice.

### 3.11. Apoptosis Detection

The ApopTag Peroxidase *in Situ* Apoptosis Detection Kit (Merck Millipore, Darmstdt, Germany) detects apoptotic cells *in situ* by labeling and detecting DNA strand breaks by the TUNEL method. The apoptosis assay was performed following the manufacturer’s instructions to detect cell death in the SW620 cell line induced by PDT treatment. For the assay, cells were prepared as described in Section 3.6. The experiment was stopped by fixation of adherent cells 24 h after PDT in 1% formaldehyde in PBS 7.4 pH. After two washes with PBS, cells were incubated with TdT enzyme at 37 °C for 1 h. The slides were washed three times in PBS for 1 min and incubated with anti-digoxigenin peroxidase conjugate in a humidified chamber for 30 min at room temperature (RT), followed by three rinses with PBS at room temperature. Then, slides were incubated with peroxidase substrate, next counterstained with hematoxylin, dehydrated and mounted in medium. To detect peroxidase-stained apoptotic bodies, brightfield microscopy was used. The number of TUNEL-positive cells was determined.

The rate of TUNEL-positive cells (peroxidase-positive) was determined by dividing the number of TUNEL-positive cells by the total number of cells in slides.

### 3.12. Statistical Analysis

Statistical analysis was performed using a multiple comparisons ANOVA test. Paired samples were compared using the Tukey HSD *post hoc* test. All values are presented as the mean ± 5% standard deviation (SD). *p* < 0.05 was considered statistically significant.

## 4. Conclusions

The role of the insulin-like growth factor-2 system in oxidative stress, and particularly in the PDT, is still far from being understood, but it seems that low dose PDT may result in high expression of some proteins, which, in turn, may limit or even impair its therapeutic effects. However, this assumption needs to be confirmed in further studies.

## Supplementary Materials

Supplementary Materials can be found at http://www.mdpi.com/1422-0067/16/10/23615/s1.
