# Supplementary material for: Insulin-Like Growth Factor-2 Is Induced Following 5-Aminolevulinic Acid-Mediated Photodynamic Therapy in SW620 Human Colon Cancer Cell Line
Source: Int J Mol Sci. 2015 Oct 2;16(10):23615–29. doi: 10.3390/ijms161023615 (PMC4632717; doi:10.3390/ijms161023615)
Supplement: Supplementary file 1 [file ijms-16-23615-s001.pdf]

Supplementary Information

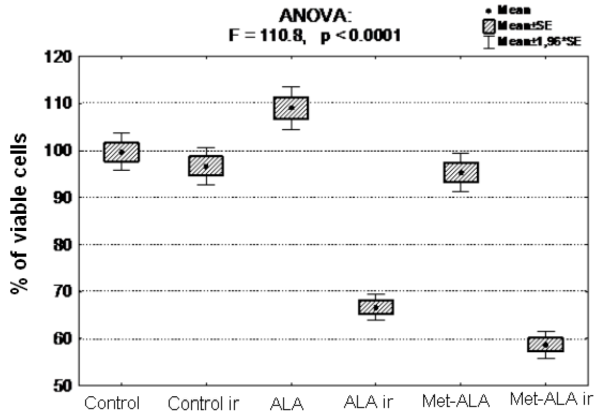

Figure S1. Percent of viable cells determined by MTT assay. All values are presented using multiple comparisons ANOVA test. Paired samples were compared using the Tukey HSD *post-hoc* test (Table S1) $p < 0.05$ was considered statistically significant. Photodynamic therapy by means of 5-ALA or its methyl ester (Met-ALA) decreased the viability of SW620 cells. Cells were treated with equal concentration of above precursors for 4 h (5-ALA, Met-ALA) and then irradiated with 4.5 J/cm^2 at 630 ± 20 nm (ir, irradiation). Cell viability was measured after 24 h following the irradiation.

Table S1. Tukey HSD *post-hoc* test of MTT assay.

**Probes**	**{1}** ***M*** **= 100**	**{2}** ***M*** **= 97**	**{3}** ***M*** **= 109**	**{4}** ***M*** **= 67**	**{5}** ***M*** **= 95**	**{6}** ***M*** **= 59**
{1} Control		$p = 0.868$	**$p = 0.043$**	**$p < 0.001$**	$p = 0.611$	**$p < 0.001$**
{2} Control ir	$p = 0.868$		**$p = 0.007$**	**$p < 0.001$**	$p = 0.996$	**$p < 0.001$**
{3} ALA	**$p = 0.043$**	**$p = 0.007$**		**$p < 0.001$**	**$p = 0.003$**	**$p < 0.001$**
{4} ALA ir	**$p < 0.001$**	**$p < 0.001$**	**$p < 0.001$**		**$p < 0.001$**	$p = 0.096$
{5} Met-ALA	$p = 0.611$	$p = 0.996$	**$p = 0.003$**	**$p < 0.001$**		**$p < 0.001$**
{6} Met-ALA ir	**$p < 0.001$**	**$p < 0.001$**	**$p < 0.001$**	$p = 0.096$	**$p < 0.001$**	

M, mean; **marked** differences are significant at $p < 0.05$.

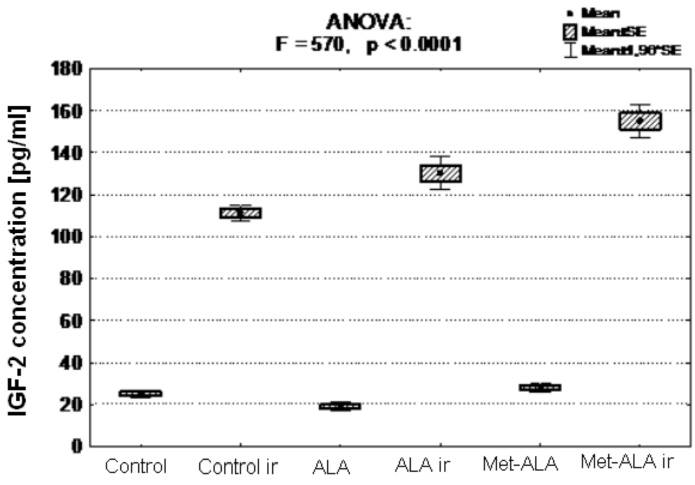

Figure S2. Results from ELISA test performed on SW620 human colon cancer cells exposed to 3 mM 5-ALA or met-ALA and 4.5 J/cm^2 light or left without a light treatment, and in control cells (no precursor, no light) or light only. A significant increase in IGF-2 concentration was found after PDT and light only (p < 0.05). Statistical analysis were preformed using multiple comparisons ANOVA test. Paired samples were compared using the Tukey HSD *post-hoc* test (Table S2) p < 0.05 was considered statistically significant. Data are presented as means with 5% statistical error from 2 experiments. ir, irradiation.

Table 2. Tukey HSD *post-hoc* test.

**Probes**	**{1}** ***M*** **= 25**	**{2}** ***M*** **= 111**	**{3}** ***M*** **= 19**	**{4}** ***M*** **= 130**	**{5}** ***M*** **= 28**	**{6}** ***M*** **= 155**
{1} Control		***p*** **< 0.001**	*p* = 0.594	***p*** **< 0.001**	*p* = 0.950	***p*** **< 0.001**
{2} Control ir	***p*** **< 0.001**		***p*** **< 0.001**	***p*** **= 0.014**	***p*** **< 0.001**	***p*** **< 0.001**
{3} ALA	*p* = 0.594	***p*** **< 0.001**		***p*** **< 0.001**	*p* = 0.257	***p*** **< 0.001**
{4} ALA ir	***p*** **< 0.001**	***p*** **= 0.014**	***p*** **< 0.001**		***p*** **< 0.001**	***p*** **= 0.004**
{5} Met-ALA	*p* = 0.950	***p*** **< 0.001**	*p* = 0.257	***p*** **< 0.001**		***p*** **< 0.001**
{6} Met-ALA ir	***p*** **< 0.001**	***p*** **< 0.001**	***p*** **< 0.001**	***p*** **= 0.004**	***p*** **< 0.001**	

M, mean; **marked** differences are significant at p < 0.05.
